# Detection of *Orientia* spp. Bacteria in Field-Collected Free-Living *Eutrombicula* Chigger Mites, United States

**DOI:** 10.3201/eid2908.230528

**Published:** 2023-08

**Authors:** Kaiying Chen, Nicholas V. Travanty, Reuben Garshong, Dac Crossley, Gideon Wasserberg, Charles S. Apperson, R. Michael Roe, Loganathan Ponnusamy

**Affiliations:** North Carolina State University, Raleigh, North Carolina, USA (K. Chen, N.V. Travanty, C.S. Apperson, R.M. Roe, L. Ponnusamy);; University of North Carolina at Greensboro, Greensboro, North Carolina, USA (R. Garshong, G. Wasserberg);; Georgia Museum of Natural History, Athens, Georgia, USA (D. Crossley)

**Keywords:** *Orientia tsutsugamushi*, *Eutrombicula*, chigger mites, scrub typhus, bacteria, vector-borne infections, parasites, North Carolina, United States

## Abstract

Scrub typhus, a rickettsial disease caused by *Orientia* spp., is transmitted by infected larval trombiculid mites (chiggers). We report the molecular detection of *Orientia* species in free-living *Eutrombicula* chiggers collected in an area in North Carolina, USA, to which spotted fever group rickettsiae infections are endemic.

Rickettsioses are distributed worldwide, caused by bacteria in the family Rickettsiaceae, genera *Orientia* and *Rickettsia* (*1*). The pathogens are transmitted by host-feeding arthropods, including ticks, mites, fleas, and lice (*2*). Among those arthropods are trombiculid mites, which have a widespread global distribution and high species diversity. Among the different stages of the lifecycle of trombiculid mites, only the larvae are ectoparasites, commonly known as chiggers (*3*). Some species are vectors of intracellular bacterial pathogens in the genus *Orientia* that causes a potentially lethal human febrile disease, scrub typhus (*4*,*5*). Scrub typhus results in considerable illness and death; it causes >1 million cases of illness each year (*5*). A recent review (*6*) concluded that trombiculid mites might be widespread vectors of other zoonotic agents not yet recognized. Spotted fever group rickettsiosis diseases, including Rocky Mountain spotted fever, have many of the same symptoms as scrub typhus. A recent review of the literature from 1997–2017 estimated >60% of the rickettsial diseases outside the United States were misdiagnosed (*7*).

Until recently, scrub typhus was exclusively reported from the so-called tsutsugamushi triangle, stretching from Pakistan in the west to far-eastern Russia in the east to northern Australia in the south. However, scrub typhus was reported recently in the Middle East, southern Chile, and Africa (*5*,*8*). The occurrence of scrub typhus pathogens in chiggers in the United States was not investigated. We report the identification of *Orientia* species in free-living chiggers collected at recreational parks in North Carolina, USA.

## The Study

In 2022, we collected free-living chiggers using the tile method (*9*) in different locations in North Carolina: we placed tiles on the ground and then visually inspected for the presence of chiggers after ≈1 minute. When chiggers were present, we collected them with forceps or a small paintbrush and transferred them into vials with 95% ethanol. We identified subsamples from each collection location based on morphological characteristics using published taxonomic keys (*10*). We identified chiggers as *Eutrombicula* on the basis of their morphology. In addition, we obtained chigger images of *Eutrombicula* using scanning electron microscopy at the Analytical Instrumentation Facility at North Carolina State University (Raleigh, North Carolina, USA) (Appendix Figure 1).

**Figure 1:**
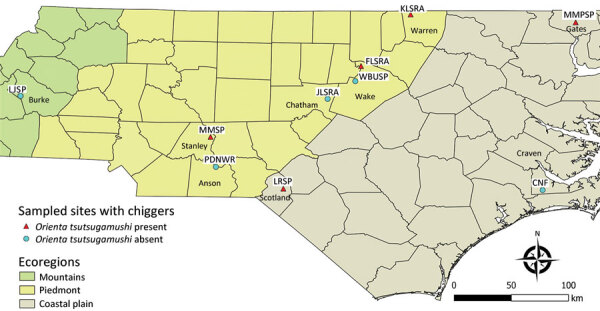
Study area for investigation of *Orientia* spp. bacteria in field-collected free-living *Eutrombicula* chigger mites, North Carolina, USA. Free-living chiggers were collected from 10 sites in 8 counties. CNF, Croatan National Forest; FLSRA, Falls Lake State Recreation Area; JLSRA, Jordan Lake State Recreation Area; KLSRA, Kerr Lake State Recreation Area; LJSP, Lake James State Park; LRSP, Lumber River State Park; MMPSP, Merchant Millpond State Park; MMSP, Morrow Mountain State Park; PDNWR, Pee Dee National Wildlife Refuge; WBUSP, William B. Umstead State Park.

We surface-sterilized individual free-living chiggers and extracted total nucleic acids using the DNeasy Blood & Tissue Kit (QIAGEN, https://www.qiagen.com) (*11*). In total, 95 chiggers from 10 different locations (10 chiggers/location; 1 location had 5 chiggers) were subjected to microbiome analyses (Figure 1). We randomly selected 8 chiggers for molecular identification using previously described 18S ribosomal RNA gene primers and PCR (*12*). Amplicons were Sanger sequenced at Eton Bioscience, Inc. (https://www.etonbio.com). The sequences (GenBank accession nos. OQ789321–5) were submitted for BLASTn (https://blast.ncbi.nlm.nih.gov/Blast.cgi) analysis and showed 99–100% identity with homologous sequences of *Eutrombicula* spp. (accession no. KY922159).

**Figure 2:**
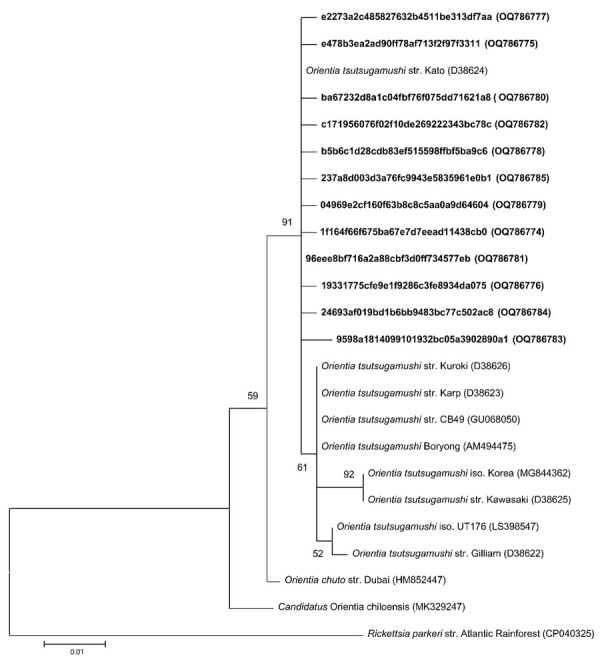
Phylogenetic tree of *Orientia tsutsugamushi* 16S rRNA gene sequences (≈399 bp) from free-living chiggers collected in North Carolina, USA, and their reference sequences in GenBank. The tree was constructed using the maximum-likelihood method. Bold text indicates study sequences. *Rickettsia parkeri* was used as an outgroup. We conducted bootstrap analyses with 1,000 iterations evaluate the strength of the tree topologies. GenBank accession numbers are in parentheses. Scale bar represents 0.01 substitutions per nucleotide position.

To determine the total bacteria present in the chiggers, we constructed a 16S rRNA sequence library of individual chiggers using the Illumina 16S rRNA (V3-V4 region) metagenomics sequencing library preparation protocol (Illumina, https://www.illumina.com) (*13*). Sequencing was performed at the University of North Carolina Microbiome Core Facility (Chapel Hill, North Carolina, USA). The Illumina FASTQ files were processed using Quantitative Insights into Microbial Ecology 2 (*14*) (Appendix). An analysis of amplicon sequence variants (ASVs) revealed that chigger mites contained reads of a bacterial sequence classified as *O. tsutsugamushi.* *O. tsutsugamushi*–positive chigger sequence reads were found in 5/10 sampling sites as follows: 3 sites in the Piedmont region (Falls Lake State Recreation Area [1 positive/10 chiggers], Kerr Lake State Recreation Area [8 positive/10 chiggers], and Morrow Mountain State Park [1 positive/10 chiggers]); and 2 sites in the Coastal Plains region (Lumber River State Park [9 positive/10 chiggers] and Merchant Millpond State Park [1 positive/5 chiggers]) (Figure 1). By using the Greengenes 16S rRNA database, we found 13 ASVs to *O. tsutsugamushi*. To further confirm those results, we extracted representative sequences from the sequencing data and conducted BLASTn searches against the National Center for Biotechnology Information (NCBI) databases. We found 12 ASVs showed a high nucleotide identity (99.5%–100%) to the *O. tsutsugamushi* strain Kato (D38624), isolated from a human scrub typhus case in Kurosawa village, Japan (https://u.osu.edu/scrubtyphus/the-kato-strain). One ASV exhibited 89.46% homology to an *O. tsutsugamushi* sequence that was excluded from the phylogenetic analysis. We then examined the approximate phylogenetic relationships for 12 of the *O. tsutsugamushi* sequence variants from our study sites with other *O. tsutsugamushi* sequences obtained from the NCBI database using BLASTn (accessed January 18, 2023). We performed multiple alignments with the ClustalW program and maximum-likelihood method tree with the Kimura 2-parameter method from the MEGA X (https://www.megasoftware.net) software package. The phylogenetic analysis revealed that all 12 of the *O. tsutsugamushi* ASVs clustered closely to *O. tsutsugamushi* strains from Asia (Figure 2).

To further verify the identity of *O. tsutsugamushi* detected in our free-living chigger samples, we amplified a 47-kDa *htrA* (high-temperature requirement A) gene in the 20 chigger samples that were positive for the *O. tsutsugamushi* 16S rRNA gene. The primers for the first round of PCR were *Ot*-145F and *Ot*-1780R, and for the second round, *Ot*-263F and *Ot*-1133R (*15*) (Appendix). The 47-kDa gene amplification products were Sanger sequenced at Eton Bioscience. Four samples (FC28, 36, 38, 109) were 92.6%–97.29% identical to the *O. tsutsugamushi* HN82 strain (GenBank accession no. LC431268) and 92%–97% identical to the *O. tsutsugamushi* Kato strain (accession no. LS398550) after trimming low-quality sequences. Sixteen samples yielded ambiguous sequences, suggesting the presence of multiple *Orientia* species or primer binding sites caused by high variation in the 47 kDa gene among *Orientia* species in our samples. Jiang et al. (*15*) studied the genetic variation of *Orientia* in this region of the genome and reported percent identity of 17 isolates of *Orientia* as 82.2%–83.3% (*15*). Among our 4 *Orientia* 47 kDa sequences from North Carolina chiggers, identity was 93.97%–98%. Phylogenetic analysis revealed that all 4 of those *O*. *tsutsugamushi* sequences clustered to *O. tsutsugamushi* strains from Asia (Appendix Figure 2).

## Conclusions

This study identified *Orientia* species within the United States in free-living *Eutrombicula* chiggers that were collected in North Carolina. This result is epidemiologically significant because it indicates vertical circulation of *Orientia* species in chiggers collected within the continental United States. The presence of *Orientia* species in free-living larvae suggests that the bacteria are maintained through transovarial transmission. Further studies are needed to complete sequencing of the 47-kDa *htrA* gene (htrA) in our samples, determine how widely distributed *Orientia* spp.–infected free-living and host-attached chiggers are in the United States, and ascertain whether wild animals that serve as hosts for chiggers become infected and infectious and develop symptoms of illness. Clinicians in this region should be alert for possible human cases of illness resulting from *Orientia* spp. infection.

Appendix Additional information about *Orientia* spp. bacteria in field-collected free-living *Eutrombicula* chigger mites, United States.
