# Supplementary material for: Detection of Orientia spp. Bacteria in Field-Collected Free-Living Eutrombicula Chigger Mites, United States
Source: Emerg Infect Dis. 2023 Aug;29(8):1676–9. doi: 10.3201/eid2908.230528 (PMC10370866; doi:10.3201/eid2908.230528)
Supplement: Appendix — Additional information about Orientia spp. bacteria in field-collected free-living Eutrombicula chigger mites, United States. [file 23-0528-Techapp-s1.pdf]

Article DOI: http://doi.org/10.3201/eid2908.230528

EID cannot ensure accessibility for supplementary materials supplied by authors. Readers who have difficulty accessing supplementary content should contact the authors for assistance.

Detection of *Orientia* spp. Bacteria in Field-Collected Free-Living *Eutrombicula* Chigger Mites, United States

Appendix

Bioinformatic Analysis of 16S rRNA Gene Sequencing Data from Free-Living Chiggers

The Illumina FASTQ files were processed using Quantitative Insights into Microbial Ecology 2 (QIIME2) (*1*). The DADA2 (*2*) plugin was used for removing noise and merging the paired-end reads, with any chimera removed. Primer sequences were trimmed, and low-quality sequences were removed by truncating the forward and reverse sequences at 280 and 220 nt, respectively. The DADA2 method replaces the traditional OTU-picking process, and instead models the sequence error to construct the actual biologic sequences present in the samples, referred to as Amplicon Sequence Variants (ASVs). The taxonomy classification was performed on the representative sequences generated from DADA2 using a Bayes classifier trained with Greengenes 13_8 at 99% identity (*3*).

Amplification of 47-kDa Gene of *Orientia* spp. from Chigger DNA

We amplified the 47 kDa gene of *Orientia* in 20 chigger samples that were positive for *Orientia* of 16S rRNA gene using nested PCR. The primers for the first round PCR were Ot-145F (5′-ACAGGCCAAGATATTGGAAG-3′) and Ot-1780R (5′-AATCGCCTTTAAACTAGATTTACTTATTA-3′). The primers for the second round PCR were Ot-263F (5′-GTGCTAAGAAARGATGATACTTC-3′)-and Ot-1133R (5′-ACATTTAACATACCACGACGAAT-3′) (*4*). In the first round of PCR mixture (10 µL) comprised 5 µL of reaction buffer, 1 µL each of forward and reverse primers at a concentration of 10 µM, 1 µL of genomic DNA, and 2 µL of sterile deionized water. For subsequent nested PCR assays (25 µL) comprised 1 µL of the amplicon from the first round of PCR was used after which 12.5 µL of reaction buffer, 1 µL each of forward and reverse primers at a concentration of

10 µM, and 9.5 µL sterile deionized water were added. Negative control reactions were included in each PCR run. The PCR amplification was performed using the following thermocycling conditions: initial denaturation at 95°C for 10 minutes, followed by 40 cycles of denaturation at 94°C for 30 seconds, annealing at 53°C for 30 seconds, and extension at 68°C for 2 minutes, with a final extension step at 72°C for 10 minutes, and a final hold at 4°C. The PCR products were subjected to Sanger sequencing at Eton Bioscience, Inc. (Research Triangle Park, NC, USA).

References

1. Bolyen E, Rideout JR, Dillon MR, Bokulich NA, Abnet CC, Al-Ghalith GA, et al. Reproducible, interactive, scalable and extensible microbiome data science using QIIME 2. Nat Biotechnol. 2019;37:852–7. PubMed https://doi.org/10.1038/s41587-019-0209-9

2. Callahan BJ, McMurdie PJ, Rosen MJ, Han AW, Johnson AJ, Holmes SP. DADA2: High-resolution sample inference from Illumina amplicon data. Nat Methods. 2016;13:581–3. PubMed https://doi.org/10.1038/nmeth.3869

3. DeSantis TZ, Hugenholtz P, Larsen N, Rojas M, Brodie EL, Keller K, et al. Greengenes, a chimera-checked 16S rRNA gene database and workbench compatible with ARB. Appl Environ Microbiol. 2006;72:5069–72. PubMed https://doi.org/10.1128/AEM.03006-05

4. Jiang J, Paris DH, Blacksell SD, Aukkanit N, Newton PN, Phetsouvanh R, et al. Diversity of the 47-kD HtrA nucleic acid and translated amino acid sequences from 17 recent human isolates of *Orientia.* Vector Borne Zoonotic Dis. 2013;13:367–75. PubMed https://doi.org/10.1089/vbz.2012.1112

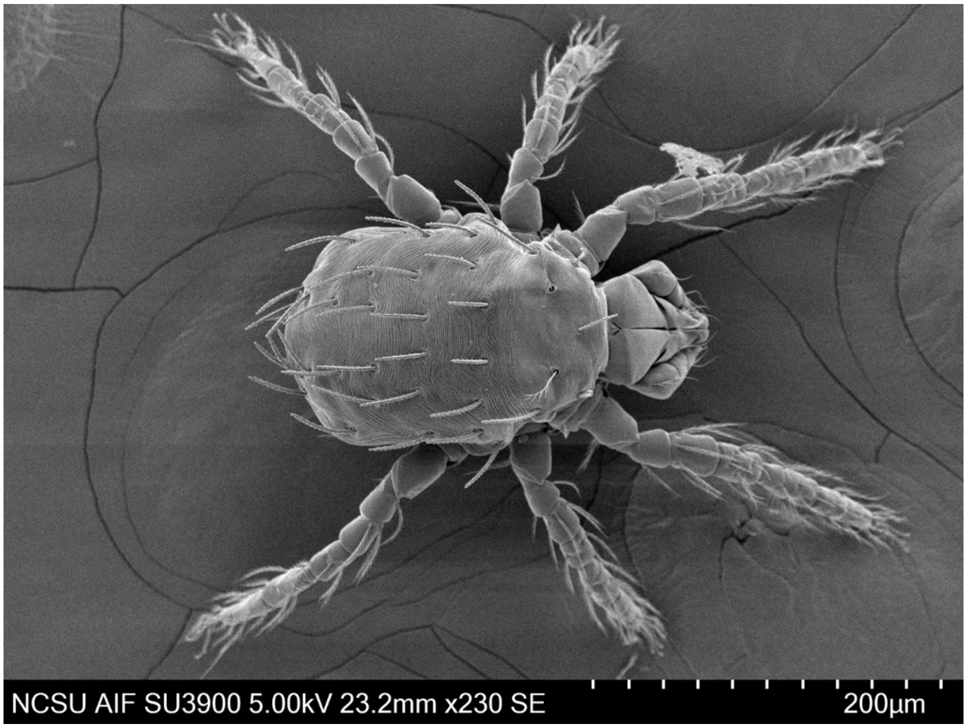

Appendix Figure 1. Scanning electron micrograph of a *Eutrombicula* chigger.

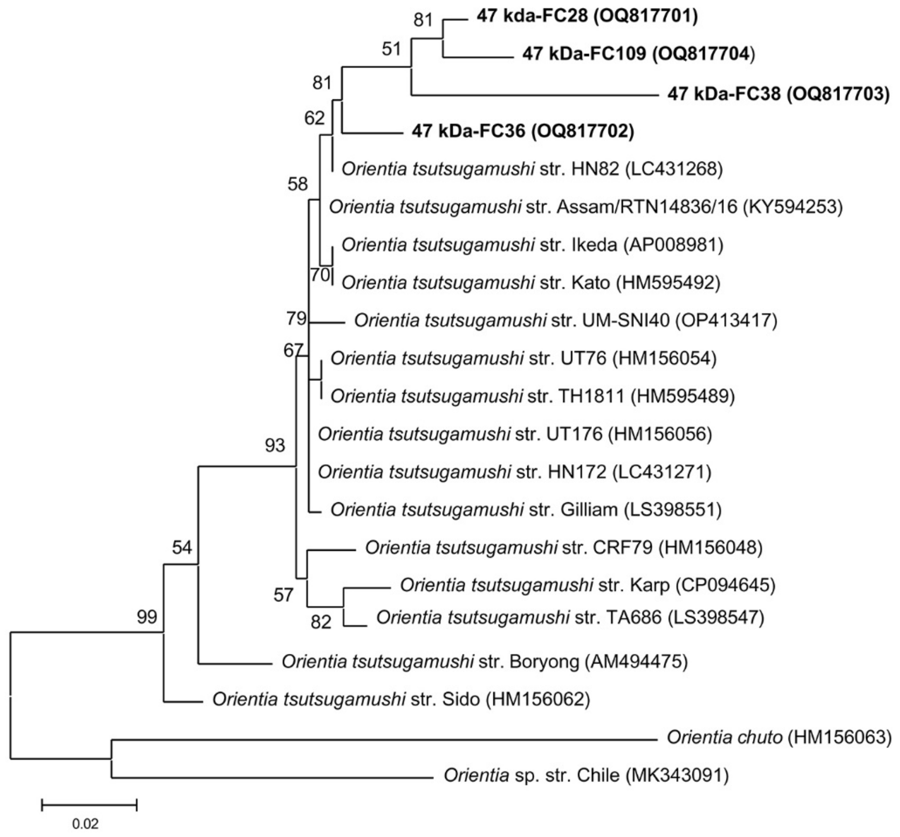

Appendix Figure 2. Phylogenetic tree of *Orientia* spp. 47 kDa sequences (≈400 bp) from free-living chiggers collected in North Carolina, USA (bold text), and their reference sequences in GenBank (in parentheses). The tree was constructed using the maximum-likelihood method. To evaluate the strength of the tree topologies, bootstrap analyses were conducted with 1,000 iterations. Scale bar represents 0.02 substitutions per nucleotide position.
